# Genetic epidemiology of Scheuermann's disease: Heritability and prevalence over a 50-year period

**DOI:** 10.3109/17453674.2011.618919

**Published:** 2011-11-24

**Authors:** Frank Damborg, Vilhelm Engell, Jan Nielsen, Kirsten O Kyvik, Mikkel Ø Andersen, Karsten Thomsen

**Affiliations:** ^1^Department of Orthopaedic Surgery, Lillebaelt Hospital, Kolding; ^2^Section of Children's Orthopaedics, Department of Orthopaedic Surgery, Odense University Hospital; ^3^Research Unit of Clinical Epidemiology, University of Southern Denmark and Center for National Clinical Databases, South, Odense University Hospital; ^4^Odense Patient Data Explorative Network (OPEN), Odense University Hospital and Institute of Regional Health Services Research, University of Southern Denmark, Odense; ^5^Spine Section, Department of Orthopaedic Surgery, Lillebaelt Hospital, Middelfart; ^6^Spine Section, Hamlet Hospital, Copenhagen, Denmark

## Abstract

**Background and purpose:**

The genetic/environmental etiology of Scheuermann's disease is unclear. We estimated the heritability of the disease using an etiological model adjusted for sex and time of diagnosis, and examined whether the prevalence of Scheuermann's disease was constant over time.

**Methods:**

46,418 twins were sent a questionnaire about health and disease. Of these, 75% returned the questionnaire and 97% answered the question “Have you been diagnosed as having Scheuermann's disease by a doctor?”

**Results:**

Responders included 11,436 complete pairs of twins. Data were analysed using classical twin modeling methods. Tetrachoric correlations were used to decide which etiological model to fit. The best-fitting model was the AE model. Heritability was 0.74 (95% CI: 0.65–0.81), while variance explained by environmental factors was 0.26 (95% CI: 0.19–0.35). A threshold of 2.1 (95% CI: 1.9–2.2) was calculated, corresponding to a prevalence of 1.9% (95% CI: 1.3–2.8) for women. Regression coefficients for age and sex were 0.000 (95% CI: –0.003 to 0.002) and –0.32 (95% CI: –0.42 to –0.23).

**Interpretation:**

We found a heritability of 0.74 in Scheuermann's disease. The threshold in men was lower than in women, corresponding to a male prevalence that was almost twice that of females. We found no change in the prevalence of Scheuermann's disease throughout the 50-year age span that we examined.

There is a considerable debate regarding the pathogenesis, natural history, and treatment of Scheuermann's disease (Tsirikos 2009). For a long time, it has been questioned whether a genetic etiology (Halal et al. 1978, Findlay et al. 1989, McKenzie and Sillence 1992, Axenovich et al. 2001, Graat et al. 2002) or an environmental etiology (Scheuermann 1920, Sørensen 1964, Lowe 1990) is most important. We have already reported results on the crude prevalence, concordance, and heritability of Scheuermann's disease based on a study of twins—with evidence of a major genetic contribution to the etiology (Damborg et al. 2006).

Twin studies are advantageous in determining whether or not the etiology of a disorder is genetic. Monozygotic (MZ) twins have identical segregating genes while dizygotic (DZ) twins, like siblings, have about 50% gene identity. Both MZ and DZ twin pairs share a common environment, which is why only genetic factors can account for a higher similarity in MZ twins than in DZ twins.

We calculated the heritability of Scheuermann's disease using an etiological model adjusted for age and sex, and examined whether the prevalence of Scheuermann's disease has been constant over the designated period of time.

## Methods

The Odense-based Danish Twin Registry (DTR) contains data on 76,000 twin pairs born in Denmark over the last 130 years. It was the first population-based twin registry to be established, and today it is one of the largest and most comprehensive twin registries in the world (Harvald et al. 2004). The zygosity of the twins in the registry is based on 4 questions of similarity, and has an accuracy of more than 95% (Magnus et al. 1983, Christiansen 2003).

Tetrachoric correlations (Hopper 1998) and estimation of heritability and the best-fitting etiological model were done using univariate structural equation modeling by using the MX software computer program (Neale et al. 2002). These calculations were based on a liability threshold model, which assumes that the dichotomous distribution of Scheuermann's disease (affected versus not affected) reflects an underlying normally distributed liability in the population. When a threshold value of the liability is exceeded, an individual is affected, otherwise not. Different thresholds in different groups, e.g. the two sexes or siblings versus unrelated individuals, reflect different loads of genetic and environmental risk factors in the groups, and thereby also the prevalence of the trait. Adjustment for age and sex effects was done through a probit regression model on the thresholds. These are standard assumptions in quantitative genetic analysis of categorical traits (Falconer and Mackay 1996). Structural equation modeling quantifies sources of individual variation by decomposing the observed phenotypic variance into genetic and environmental variance (Neale et al. 2002). The genetic contribution can be further divided into an additive (A) genetic variance component (representing the influence of alleles at several loci acting in an additive manner) and a non-additive (D) genetic variance component (representing intra- and inter-locus interaction). The environmental component is subdivided into a common (C) environmental variance component (representing environmental factors affecting both twins in a pair, and a source of similarity) and an individual/unique environmental variance component (E) (environmental factors not shared by twins and making them dissimilar). The latter also contains measurement error. Heritability is defined as the proportion of the total phenotypic variance that is attributable to genetic variance.

The tetrachoric correlations are used to decide which etiological model to fit. Selection of the best-fitting sub-model is based on a balance between goodness of fit and parsimony (Neale et al. 2002).

SPSS software version 6, Epi-Info, and MX were used. All the scientific-ethical committees of Denmark approved the study (case file 20010202).

## Materials

All 46,418 Danish twins registered in the DTR (born between 1931 and 1982) received a 17-page Omnibus questionnaire in the spring of 2002. One question was “Have you been diagnosed as having Scheuermann's disease by a doctor?”.

## Results

34,944 subjects (75%) responded to the questionnaire and 34,007 (97%) of them answered the question about Scheuermann. 943 twin individuals (380 females and 563 males) reported having Scheuermann's disease, corresponding to an overall prevalence of 2.8% (95% CI: 2.6–3.0). The prevalence was 2.1% (CI: 1.9–2.3) in females and 3.6% (CI: 3.2– 4.1) in males (chi-square; p < 0.001). The prevalence of Scheuermann's disease was not statistically significantly different between monozygotic (MZ) and dizygotic (DZ) twins. Of the above 34,007 individuals, there were 11,436 twin pairs where both twins answered the question, and of these pairs, 645 pairs reported having been diagnosed with Scheuermann's disease.

The tetrachoric correlation in MZ twins was twice as high as in DZ twins: 0.73 (CI: 0.64–0.81) as opposed to 0.33 (CI: 0.22–0.43), and this indicated that an AE sub-model should fit the data. There was no evidence of sex differences on the tetrachoric correlations (p = 0.8). We therefore continued with a joint genetic model for men and women combined. The heritability derived from the AE sub-model was 0.74 (CI: 0.65–0.81) and the environmental factor accounted for 0.26 (CI: 0.19–0.35) of the variance (Table).

**Table:** Tetrachoric correlations used to decide which etiological model to fit. Selection of the best-fitting sub-model is based on a balance between goodness of fit and parsimony. Here the AE model is the “best-fitting model”

	A	D	C	E	AIC	p-value **^a^**	p-value **^b^**
ACE	0.74 (0.37–0.81)		0.00 (0.00–0.32)	0.26 (0.19–0.36)	–25485.44		
ADE	0.74 (0.26–0.81)	0.00 (0.00–0.49)		0.26 (0.19–0.35)	–25485.44		
AE	0.74 (0.65–0.81)			0.26 (0.19–0.35)	–25487.44	0.99	1.00
DE		0.75 (0.66–0.82)		0.25 (0.18–0.34)	–25479.42		0.01
CE			0.57 (0.49–0.65)	0.43 (0.35–0.51)	–25468.82	< 0.01	
E				1.00 (1.00–1.00)	–25327.80	< 0.01	< 0.01

**^a^** comparing sub-model to ACE model.

**^b^** comparing sub-model to ADE model.

We calculated a threshold of 2.1 (CI: 1.9–2.2) for women and 1.8 (CI: 1.6–1.9) for men. This threshold corresponded to a prevalence of 1.9% (CI: 1.3–2.8) for women. Regression coefficients for age and sex effects on the thresholds were calculated. The regression coefficients were 0.0 (95% CI: –0.003 to 0.002) for age and –0.32 (95% CI: –0.42 to –0.23) for sex. The negative regression coefficient for sex meant a lower threshold for men than for women, and hence a higher male prevalence. The calculated male prevalence was 4.0% (CI: 2.8–5.7).

## Discussion

The reported prevalence of Scheuermann's disease in the general population ranges from 1% to 8% (Sørensen 1964, Scholes et al. 1991). We regard the observed self-reported prevalence of 2.8% in this large cohort as a reliable figure for the overall prevalence of symptomatic Scheuermann's disease.

The gender-related prevalence of Scheuermann's kyphosis varies in the literature. The male to female ratio has been reported to be 1:2 (Bradford et al. 1974), 1:1 (Montgomery et al. 1981, Schufflebarger and Clark 1992, Tribus 1998), 2:1 (Fisk et al. 1984, Murrey et al. 1993), or 7.3:1 (Scheuermann 1920). In our study, the male prevalence was found to be almost twice that of the female prevalence.

The estimated prevalence in males and females based on the heritability and the threshold and regression coefficient for gender gave a calculated male prevalence of 4% and a calculated female prevalence of 2%. These calculated values correspond to a ratio of 2 males to 1 female.

There have been no reports in the literature on an increased or a decreased risk of Scheuermann's disease in twins, and we found similar prevalence for MZ and DZ twins. We therefore regard the observed and calculated values of prevalence to be good and valid estimates of the true values in symptomatic Scheuermann's disease.

Twin studies are accepted as being fairly unique in discriminating the contribution of genetic factors from that of environmental factors in the phenotypic variance. It is essential that the twin cohort is population–based, since disease-based studies have a tendency to overrepresent MZ and concordant pairs (Harvald and Hauge 1965, Gedda 1966). The present cohort from the Danish Twin Registry is both very large and population-based. This optimizes the premises for this type of study. The zygosity was determined by questionnaire, which is feasible for studies of this size, and the frequency of misclassification is no higher than 5% (Magnus et al. 1983, Christiansen 2003).

The equal environment assumption is crucial for twin studies. This has been questioned repeatedly, and while there is no doubt that MZ twins are treated more similarly than DZ twins, it is questionable whether this environmental similarity causes increased phenotypic concordance. Environmental similarity during childhood is not predictive of twin similarity in personality, attitudes, or a range of psychiatric disorders, so we do not think that it would predict Scheuermann's disease similarity (Evans and Martin 2000).

The main challenge was identification of the patients. Although this was done using a questionnaire, the case definition was not simply self-reported since the disorder had to be diagnosed by a doctor. It should therefore be kept in mind that the patients would have to have experienced symptoms or to have had cosmetic complaints in order to seek medical help. Firstly, the patients would have to recall that they had been diagnosed as having Scheuermann's disease; secondly, patients with unrecognized kyphosis may not have sought medical advice and the diagnosis would not have been made; thirdly, some of these patients were born in the 1930s and 1940s, and for those patients the diagnosis may not have been made—since Scheuermann's disease was first described in 1920 (Scheuermann 1920) and may not have been well-known or accepted at the time. There is therefore a risk that cases with no symptoms or cosmetic complaints would be underestimated in this study. This means that the prevalence estimates are probably conservative.

We also calculated a regression coefficient of age. Since the questionnaire was cross-sectional, the information about Scheuermann's disease does not reflect the age at which the different individuals had their diagnosis. It merely tells us who has been diagnosed with the disease in the time in question, and it therefore tells us whether there is a tendency for the diagnosis to become more or less frequent over time.

The fact that we found a regression coefficient for age of 0, with a very narrow 95% confidence interval (–0.003 to 0.002), impliesndicates that there had been no changes in the prevalence of Scheuermann's disease over the 50-year age span that we examined.

Different theories mainly based on genetic factors (Halal et al. 1978, Findlay et al. 1989, McKenzie and Sillence 1992, Axenovich et al. 2001, Graat et al. 2002) and mechanical factors (Scheuermann 1920, Sørensen 1964, Lowe 1990) have been proposed to explain the etiology in Scheuermann's kyphosis.

The first group to report a possible mode of autosomal dominant inheritance was Kewalramani et al. (1976), who found this in a family with Scheuermann's kyphoscoliosis in combination with the Charcot-Marie-Tooth syndrome. Halal et al. (1978) supported this theory by describing 5 families in which the prevalence of Scheuermann's kyphosis appeared to follow an autosomal dominant pattern. Later studies supported this (Findlay et al. 1989, McKenzie and Sillence 1992). Axenovich et al. (2001) published a study on 90 pedigrees with Scheuermann's disease and via the transmission probability model they concluded that the inheritance could be described within the framework of a dominant major gene diallele model. According to this model, Scheuermann's disease should never occur in the absence of the mutant allele—so that all of the males as opposed to only half of the female carriers of the mutant allele would develop the disease.

Our results are mainly in accordance with a multifactorial inheritance pattern of Scheuermann's disease, but we cannot rule out that a major gene might be involved in the etiology. Our values of the male to female ratio of approximately 2 males to 1 female could be seen to substantiate the above-mentioned theory, where Scheuermann's disease should never occur in the absence of the mutant allele so that all the male carriers as opposed to only half the female carriers of the mutant allele develop the disease, giving a male–to-female factor of 2.

We believe that the estimate of the heritability of Scheuermann's disease, threshold values, and regression coefficients provided by this study will be valuable in the design of future studies to identify underlying genes.
